# Vitamin B6 Modifies Tau-Induced Cellular Stress- and Proteostasis-Related Responses

**DOI:** 10.3390/jof12090662

**Published:** 2026-09-02

**Authors:** Merve Yılmazer

**Affiliations:** Department of Molecular Biology and Genetics, Faculty of Science, Istanbul University, 34134 Istanbul, Turkey; merve.yilmazer@istanbul.edu.tr

**Keywords:** cellular stress response, fission yeast, protein tau, pyridoxal 5′-phosphate, vitamin B6

## Abstract

Abnormal tau expression is associated with disruption of cellular homeostasis and activation of stress-response pathways. However, the effects of vitamin B6 on cellular stress responses associated with tau expression remain unclear. In this study, the influence of vitamin B6 on oxidative stress responses, endoplasmic reticulum (ER) stress and unfolded protein response (UPR) signaling, autophagy-related gene expression, and apoptosis-related responses was investigated using a human tau-expressing *Schizosaccharomyces pombe* model. Cells expressing human tau were treated with pyridoxal 5′-phosphate (PLP), the biologically active form of vitamin B6, and changes in oxidative stress responses, endoplasmic reticulum (ER) stress and unfolded protein response (UPR) signaling, autophagy-related gene expression, apoptosis-related responses, and protein carbonyl content were evaluated. Tau expression was associated with increased expression of oxidative stress-, ER stress-, and autophagy-related genes. Vitamin B6 altered several of these responses, including the expression of ER stress- and oxidative stress-related genes. Although intracellular ROS levels increased following vitamin B6 treatment, protein carbonyl levels remained largely unchanged. In contrast, apoptosis-related responses showed only limited changes. These findings show that vitamin B6 is associated with changes in selected stress- and proteostasis-related responses in tau-expressing cells. The observed effects were not uniform across all pathways, indicating a complex relationship between vitamin B6, tau expression, and cellular stress responses. This study provides further insight into cellular responses associated with tau expression and supports further investigation of vitamin B6-mediated effects in more complex experimental models.

## 1. Introduction

Tau protein aggregation is a defining feature of several neurodegenerative disorders and is closely associated with cellular dysfunction. Beyond its physiological role in microtubule stabilization, aberrant tau accumulation disrupts proteostasis and triggers multiple cellular stress responses, including oxidative stress and impaired protein quality control. Increasing evidence suggests that tau-induced toxicity is not solely driven by protein aggregation itself but also by the inability of cellular stress-response pathways to maintain homeostasis. Tau is a microtubule-associated protein encoded by the human *MAPT* gene, and abnormal tau metabolism leads to its accumulation and the formation of neurofibrillary tangles, a pathological hallmark of tauopathies such as Alzheimer’s disease [1,2]. Tau aggregation is largely promoted by hyperphosphorylation at multiple sites, resulting in conformational changes that reduce its affinity for microtubules. Importantly, many kinases involved in tau phosphorylation, including glycogen synthase kinase 3, are highly conserved across eukaryotes, supporting the use of simple model organisms to investigate conserved mechanisms underlying tau-associated cellular dysfunction [3].

Simple eukaryotic model organisms have proven valuable for dissecting conserved mechanisms associated with protein aggregation and cellular toxicity. Yeast models, in particular, provide genetically tractable systems for studying cellular stress responses relevant to human disease. *Schizosaccharomyces pombe* represents a powerful model organism because many of its stress signaling pathways, protein quality control mechanisms, and post-translational regulatory processes are conserved in higher eukaryotes. In addition, *S. pombe* has been widely used to investigate endoplasmic reticulum (ER) stress and unfolded protein response (UPR) pathways, providing important insights into cellular stress adaptation [4,5]. These characteristics make *S. pombe* a suitable system not only for studying fundamental aspects of eukaryotic cell biology but also for modeling conserved molecular mechanisms associated with disease-related proteotoxic stress [6,7,8]. Expression of human tau in yeast has been shown to induce proteotoxic stress and activate stress-responsive pathways, supporting the utility of yeast models for investigating cellular consequences of tau expression [9,10].

Human tau-expressing *S. pombe* has previously been established as a model for investigating tau-associated cellular processes. In our recent work, we demonstrated that vitamin B6 influences *MAPT* expression as well as tau protein levels and phosphorylation status in this model [11]. These findings suggested a potential interaction between vitamin B6 and tau-associated cellular pathways. However, the effects of vitamin B6 on cellular stress responses and proteostasis-related mechanisms associated with tau expression remain largely unexplored.

Endoplasmic reticulum (ER) stress arises from the accumulation of misfolded or unfolded proteins within the ER lumen and triggers activation of the unfolded protein response, a conserved adaptive mechanism that aims to restore protein homeostasis [12]. Increasing evidence indicates that ER stress is closely linked to tau protein homeostasis and contributes to cellular dysfunction associated with tau pathology. Pathological forms of tau, particularly hyperphosphorylated tau, have been reported to impair protein folding capacity and activate ER stress-associated signaling pathways, suggesting a reciprocal relationship between tau accumulation and ER stress responses [13,14]. Persistent ER stress and dysregulated UPR signaling have been implicated in several neurodegenerative disorders, including Alzheimer’s disease, where proteostatic imbalance is a prominent pathological feature [15]. Therefore, ER stress responses represent an important component of tau-associated cellular dysfunction and a relevant target for investigating factors that may modify cellular adaptation to proteotoxic stress.

Vitamin B6 is a water-soluble vitamin that exists in three interconvertible forms: pyridoxine, pyridoxal, and pyridoxamine. Its biologically active form, pyridoxal 5′-phosphate (PLP), functions as a coenzyme in numerous metabolic reactions and plays essential roles in amino acid metabolism, neurotransmitter synthesis, redox balance, and cellular homeostasis [16,17]. In addition to its metabolic functions, vitamin B6 has been reported to contribute to cellular defense mechanisms by limiting reactive oxygen species accumulation and modulating stress-related pathways [18]. Altered vitamin B6 status has been associated with several neurological and neurodegenerative disorders, including epilepsy, autism spectrum disorders, Down syndrome, schizophrenia, Alzheimer’s disease, and Parkinson’s disease, and reduced plasma PLP levels have been reported in affected individuals [19,20].

Recent experimental evidence has begun to link vitamin B6 status with tau-related pathological changes. In mice, dietary vitamin B6 restriction reduced brain PLP levels and increased markers associated with oxidative stress, ER stress, apoptotic cell death, amyloid-β deposition, and tau hyperphosphorylation [21]. More recently, vitamin B6 treatment reduced GSK-3β Tyr216 phosphorylation, tau hyperphosphorylation, and amyloid-β production in DAB-treated SH-SY5Y cells [22]. In our recent study using tau-expressing fission yeast, PLP treatment also reduced MAPT expression, total tau levels, and tau phosphorylation at S262, S396, and S404 [11]. Although these findings indicate a relationship between vitamin B6 status and tau-related changes, direct evidence remains limited, and its effects on cellular stress- and proteostasis-related responses associated with tau expression are not fully understood.

While our previous study [11] focused primarily on the metabolic responses of vitamin B6 in human tau-expressing *S. pombe* cells, the effects of vitamin B6 on tau-associated cellular stress responses and protein quality control mechanisms remain unclear. Therefore, the present study investigated whether vitamin B6 modifies cellular stress adaptation in *S. pombe* cells expressing human tau protein. Particular attention was given to oxidative stress responses, protein carbonylation, apoptosis- and autophagy-related pathways, and ER stress-associated unfolded protein response signaling, including GRP78 and Ire1 expression. By examining multiple components of the cellular stress network, this study aimed to determine whether vitamin B6 influences the cellular consequences of tau expression and contributes to the regulation of proteostasis-related responses in a simple eukaryotic model system.

## 2. Materials and Methods

### 2.1. Yeast Strain, Culture Conditions, and Vitamin B6 Treatment

In this study, *S. pombe* pMS cells carrying the empty vector and pMS-*mapt* cells expressing human tau protein were used [23]. Cells were cultured in minimal medium supplemented with 1% glucose and 50 mg/L guanine and incubated at 30 °C with shaking at 180 rpm. All experiments were initiated at a cell density of 1 × 10^6^ cells/mL.

To repress tau expression, thiamine was added to the culture medium at a final concentration of 15 µM, and cells were maintained under repressing conditions for 24 h. Tau expression was subsequently induced by thiamine removal, followed by incubation for an additional 20 h. After induction, cells were treated with pyridoxal 5′-phosphate (PLP) at a final concentration of 200 µM for 4 h. The PLP concentration and treatment period were selected based on dose- and time-dependent optimization experiments reported in our previous study, in which these conditions were shown to modulate tau-associated cellular responses [11]. At the end of the treatment period, cells were harvested and used for all subsequent analyses.

### 2.2. Analysis of Stress-, Autophagy-, and Apoptosis-Related Gene Expression

Total RNA was isolated from vitamin B6 (200 µM PLP)-treated and untreated *S. pombe* cells grown under tau protein-producing conditions using the Invitrogen PureLink™ RNA Mini Kit (Thermo Fisher Scientific Baltics UAB, Vilnius, Lithuania), according to the manufacturer’s instructions. Following RNA isolation, complementary DNA (cDNA) was synthesized using the Applied Biosystems™ High-Capacity cDNA Reverse Transcription Kit (Thermo Fisher Scientific Baltics UAB, Vilnius, Lithuania).

To investigate the effects of vitamin B6 on tau-associated cellular stress responses, the expression levels of genes involved in oxidative stress (*sod1*, *ctt1*), endoplasmic reticulum stress and unfolded protein response (*ire1*, *bip1*(homologous of human *GRP78*), *gpt1*, *hsp90*), apoptosis-related pathways (*aif1*), and autophagy-related pathways (*atg6*, *atg14*, *trs85*) were analyzed. In addition, *MAPT* gene expression was evaluated to confirm the effects of PLP treatment under the experimental conditions (1% glucose concentration) used in this study (Supplementary Figure S1). Gene expression levels were normalized to the reference gene *act1*, which has been used as an internal reference in real-time quantitative PCR (RT-qPCR) studies conducted in *S. pombe* [10,24,25]. Untreated pMS cells carrying the empty vector were used as the calibrator group, and their relative expression level was set to 1. Melting-curve analysis was performed after amplification to assess product specificity, and a single melting peak was observed for each primer pair.

RT-qPCR was performed using a Roche LightCycler^®^ 480 system (Roche Diagnostics GmbH, Mannheim, Germany) with SYBR Green-based detection and PowerUp™ SYBR™ Green Master Mix (Thermo Fisher Scientific Baltics UAB, Vilnius, Lithuania). PCR amplification was carried out according to the parameters specified by the manufacturer. Melting-curve analysis was performed after each amplification run, and a single melting peak was observed for each primer pair. Relative gene expression levels were analyzed using the Pfaffl method [26]. All experiments were performed in three independent biological replicates.

### 2.3. Western Blot Analysis

Total protein extraction was performed from *S. pombe* cells using the method described by Zhang et al. (2001) [27]. Cells were resuspended in lysis buffer containing 5 mM EDTA, 50 mM Tris–HCl, 0.5% Nonidet P-40, and 150 mM sodium chloride, followed by mechanical disruption with glass beads (0.45–0.50 mm diameter) using a cell disruptor. Protein concentrations were determined using the Smart BCA Protein Assay Kit (iNtRON Biotechnology, Inc., Seongnam, Republic of Korea) according to the manufacturer’s instructions.

Equal amounts of total protein (30 µg) were separated by 10% SDS–polyacrylamide gel electrophoresis (SDS–PAGE) and subsequently transferred onto a PVDF membrane (Immun-Blot^®^ PVDF Membrane, Bio-Rad Laboratories, Singapore). To confirm the effects of PLP treatment on tau expression under the experimental conditions used in the present study, total tau and phosphorylated tau levels were evaluated using antibodies against phosphorylated tau at serine residues S262 (Abclonal Phospho-Tau-S262, 1:1000), S396 (Abcam anti-phospho-tau Ser396 [E178], 1:1000), and S404 (Abclonal Phospho-Tau-S404 Rabbit pAb, 1:1000), as well as total tau protein (Proteintech anti-tau rabbit IgG 10274-1-AP, 1:1000) (Supplementary Figure S1).

To assess ER stress-associated signaling, membranes were also probed with antibodies against GRP78/HSPA5 (Novus Biologicals Rabbit GRP78/HSPA5 antibody, 1:2500) and IRE1α (Novus Biologicals Rabbit IRE1α antibody, 1:1000). For normalization, α-tubulin (Abclonal α-Tubulin Rabbit mAb (A6830), 1:1000) and GAPDH (Anti-GAPDH mouse IgG, MA5-15738, Invitrogen, Carlsbad, CA, USA; 1:2500) were used as internal loading controls. Target proteins and their corresponding loading controls were detected on different membranes processed simultaneously under identical experimental conditions. Equal amounts of protein (30 µg) from the same biological samples were loaded onto each membrane in the same sample order. Following primary antibody incubation, membranes were treated with appropriate horseradish peroxidase (HRP)-conjugated secondary antibodies, including goat anti-rabbit IgG (Abclonal HRP-conjugated Goat anti-Rabbit IgG (H+L) (AS014), 1:1000) and HRP conjugated anti-mouse IgG (BA1050, 1:5000). Immunoreactive bands were visualized using enhanced chemiluminescence (GLPBIO Ultra High Sensitivity ECL Kit, GLPBIO Technology LLC, Montclair, CA, USA), and immunoblot signals were captured with a ChemiDoc XRS imaging system (Bio-Rad Laboratories, Inc., Hercules, CA, USA). Band intensities were analyzed using Image Lab software version 6.0.1 (Bio-Rad, Hercules, CA, USA).

### 2.4. Measurement of Intracellular Reactive Oxygen Species (ROS)

Intracellular ROS production was assessed by measuring the fluorescence of 2′,7′-dichlorofluorescein diacetate (DCF-DA), as described previously [28,29]. Briefly, cells were incubated with DCF-DA at a final concentration of 40 µM at 30 °C for 1 h. Following incubation, cells were washed to remove excess dye.

Fluorescence measurements were recorded kinetically for approximately 2.5 h using a spectrofluorometer (Bio-Tek FLx800, BioTek Instruments, Inc., Winooski, VT, USA) with excitation and emission wavelengths set at 490 nm and 524 nm, respectively. ROS production was evaluated from the rate of increase in fluorescence over time, and the slope values obtained from the kinetic fluorescence curves were used for statistical analyses.

### 2.5. Determination of Apoptosis Rate by Fluorescence Microscopy

Apoptotic cell death was evaluated using acridine orange/ethidium bromide (AO/EtBr) dual staining, as previously described [30]. Cells were washed with phosphate-buffered saline (PBS) and stained with AO/EtBr prior to analysis. The staining solution was prepared by mixing 60 µL acridine orange (1 mg/mL), 100 µL ethidium bromide (1 mg/mL), and 840 µL PBS, and 5 µL of the resulting solution was added to each cell suspension. Stained cells were examined using an Olympus BX53 fluorescence microscope (Olympus Corporation, Tokyo, Japan).

Three independent biological replicates were performed. A total of 80 fluorescence images, consisting of 40 AO-stained and 40 EtBr-stained images, were analyzed across all biological replicates. Total cell number was determined from AO-stained images, whereas EtBr-positive cells were considered apoptotic. The percentage of apoptotic cells was calculated as the ratio of EtBr-positive cells to the total number of cells counted. Cell counting and image analysis were performed using ImageJ software 1.54g.

### 2.6. Protein Carbonyl Content Assay

Protein carbonyl content was measured as an indicator of oxidative protein damage using the Elabscience^®^ Protein Carbonyl Colorimetric Assay Kit (Elabscience Biotechnology Inc., Houston, TX, USA), according to the manufacturer’s instructions. Briefly, protein extracts obtained from untreated and PLP-treated cells were used for the assay. Absorbance was measured at 370 nm using a microplate reader. Protein carbonyl content was calculated according to the manufacturer’s protocol and normalized to total protein concentration. Results were expressed as nanomoles of protein carbonyls per milligram of protein (nmol/mg protein).

### 2.7. Statistical Analysis

Data are presented as mean ± standard deviation (SD). Statistical analyses were performed using GraphPad Prism version 9. The normality assumption was evaluated by visual inspection of Q–Q plots of the residuals, which showed no marked deviations from normality. Gene expression, ROS production, protein carbonyl content, apoptosis rate, and protein expression data were analyzed by two-way analysis of variance (ANOVA) to evaluate the effects of strain, PLP treatment, and their interaction, followed by Tukey’s multiple comparison test in GraphPad Prism 9. Differences were considered statistically significant at *p* < 0.05.

## 3. Results

To verify the effects of vitamin B6 under the experimental conditions used in the present study (under 1% glucose conditions), *MAPT* expression, total tau levels, and tau phosphorylation were also evaluated. Consistent with our previous findings (under 0.5% glucose conditions), vitamin B6 treatment reduced MAPT transcript levels and decreased tau phosphorylation at the examined residues (Supplementary Figure S1) [11]. Following confirmation of these effects, subsequent analyses focused on cellular stress responses and proteostasis-related pathways associated with tau expression.

### 3.1. Oxidative Stress Responses

**Figure 1:**
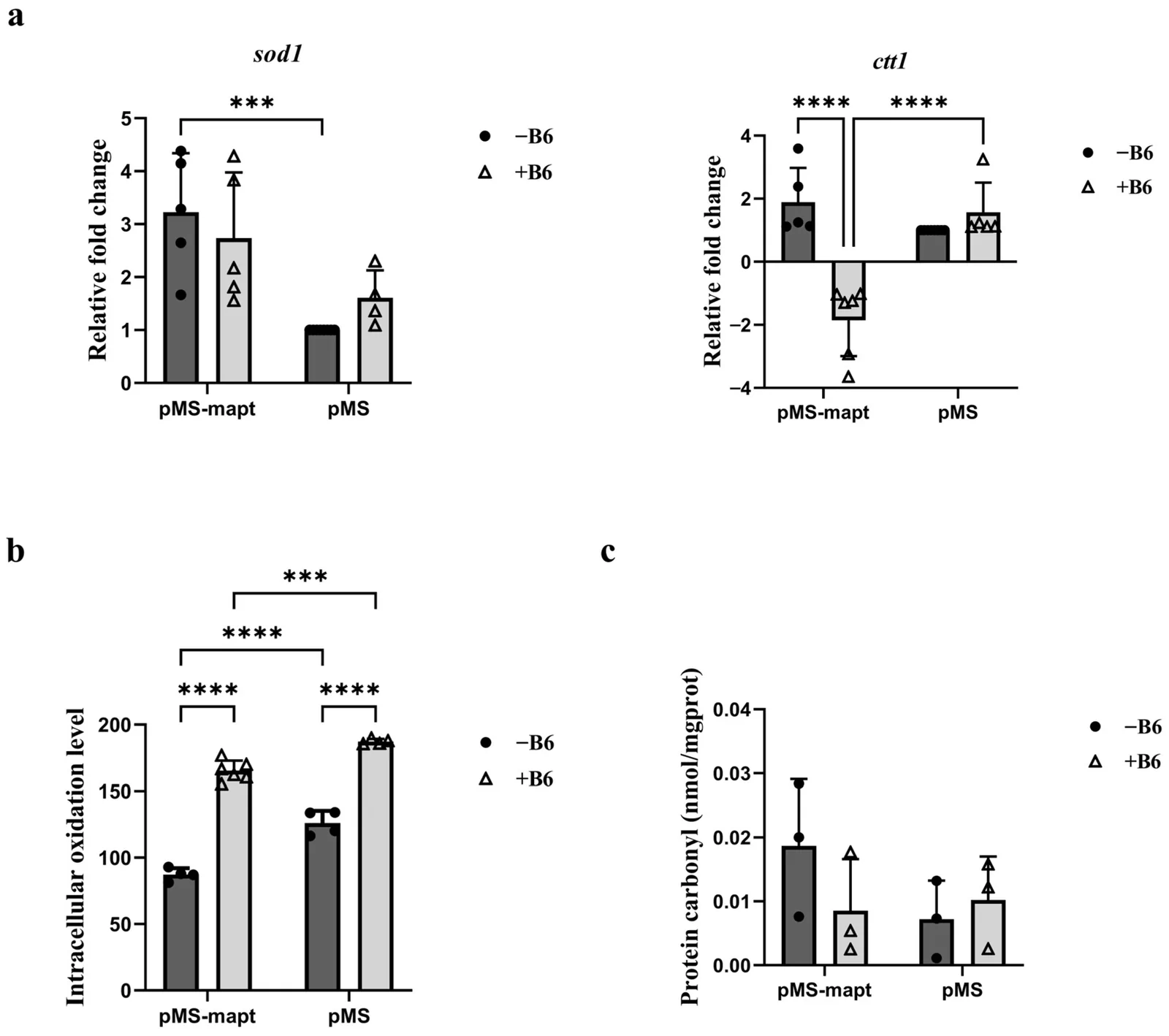
Effects of vitamin B6 on oxidative stress-related responses in tau-expressing *S. pombe* cells. (**a**) Relative expression levels of the oxidative stress-response genes *ctt1* and *sod1* in pMS and pMS-mapt cells with or without vitamin B6 treatment. Gene expression levels were normalized to *act1* and are presented relative to untreated pMS cells (control). (**b**) Intracellular ROS levels measured by DCFH-DA fluorescence. ROS levels were calculated from the max slope values of fluorescence curves. (**c**) Protein carbonyl content (nmol/mg protein) as an indicator of oxidative protein damage. Data were analyzed using two-way ANOVA and presented as mean ± SD from three independent experiments (*** *p* < 0.001, and **** *p* < 0.0001).

To investigate oxidative stress-related responses, the expression levels of the stress-response genes *ctt1* and *sod1* were analyzed (Figure 1a). Relative to the control group (pMS), untreated pMS-mapt cells exhibited increased expression of both genes, with *ctt1* and *sod1* expression elevated by 1.9-fold and 3.2-fold, respectively. In vitamin B6-treated pMS-mapt cells, *ctt1* expression was 1.86-fold lower than that of the control group, whereas *sod1* expression remained higher than control, showing a 2.73-fold increase. However, the *sod1* increase observed after vitamin B6 treatment was lower than that detected in untreated pMS-mapt cells. In pMS cells, vitamin B6 treatment increased *ctt1* and *sod1* expression by 1.57- and 1.61-fold, respectively, compared with untreated pMS cells.

Intracellular ROS production was assessed using a DCFH-DA-based fluorescence assay, and ROS levels were calculated from the max slope values of the fluorescence curves (Figure 1b). Under untreated conditions, ROS production was higher in pMS cells than in pMS-mapt cells. Vitamin B6 treatment increased ROS production in both strains, resulting in a 1.89-fold increase in pMS-mapt cells and a 1.48-fold increase in pMS cells relative to their corresponding untreated controls.

To evaluate oxidative protein damage, protein carbonyl content was determined (Figure 1c). The highest protein carbonyl level was observed in untreated pMS-mapt cells (0.0186 nmol/mg protein), whereas vitamin B6-treated pMS-mapt cells showed lower values (0.0085 nmol/mg protein). In pMS cells, protein carbonyl content was 0.0072 nmol/mg protein in untreated cultures and 0.0102 nmol/mg protein following vitamin B6 treatment. However, no statistically significant differences were detected among the experimental groups.

### 3.2. Effects of Vitamin B6 on ER Stress- and UPR-Related Responses

**Figure 2:**
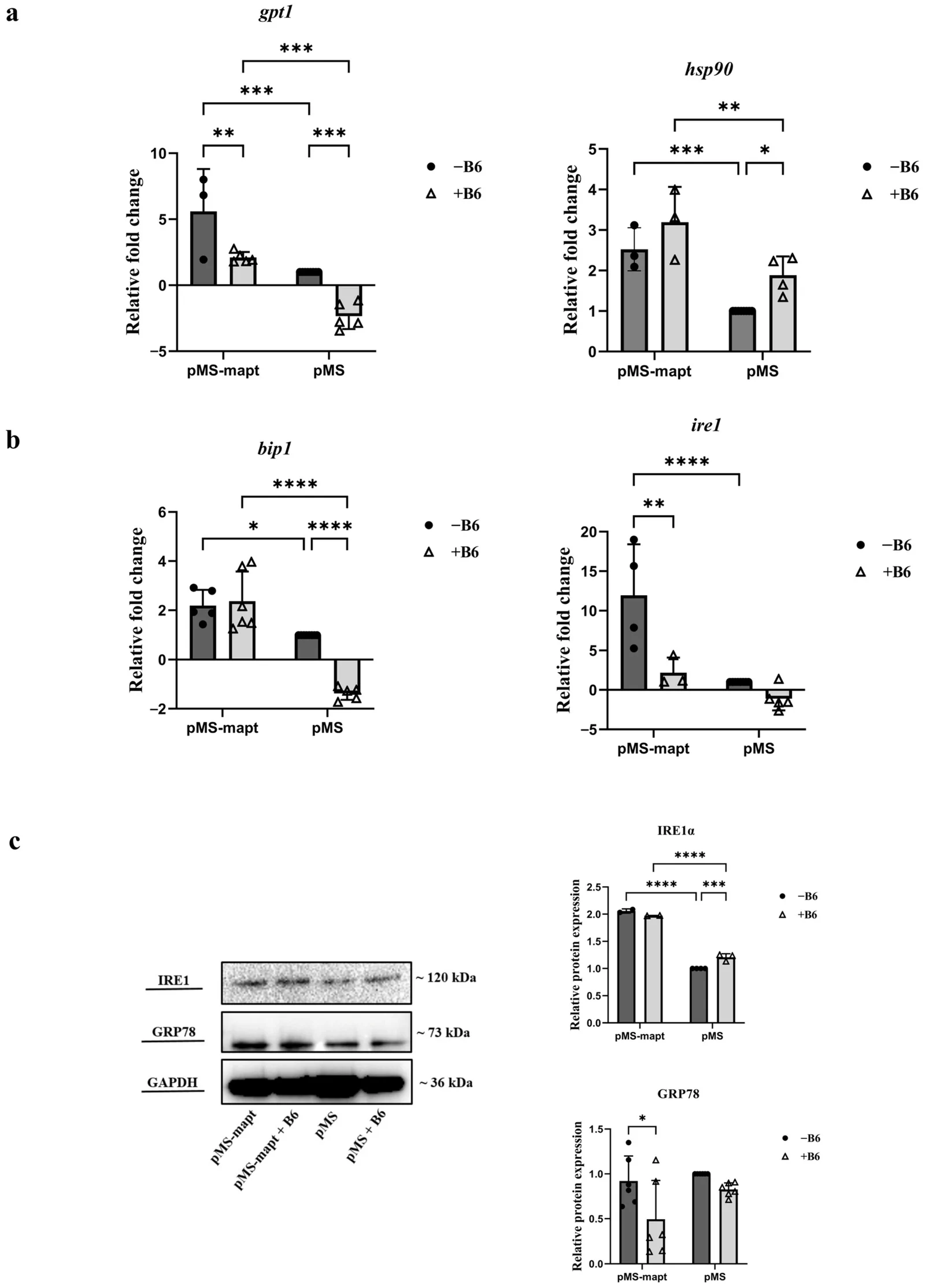
Effects of vitamin B6 on ER stress-related responses in tau-expressing *S. pombe* cells. (**a**,**b**) Relative expression levels of the ER stress-associated genes *gpt1*, *hsp90*, *bip1*, and *ire1* in pMS and pMS-mapt cells with or without vitamin B6 treatment. Transcript levels were determined by RT-qPCR, normalized to *act1*, and expressed relative to the pMS control group. (**c**) Representative Western blot analysis of IRE1α and GRP78 protein levels in pMS and pMS-mapt cells with or without vitamin B6 treatment. GAPDH was used as the loading control. Band intensities were quantified using Image Lab 6.0.1 software (Bio-Rad), normalized to GAPDH, and expressed relative to the pMS control group. Data represent as mean ± SD from three independent biological replicates. Statistical significance was determined by two-way ANOVA followed by Tukey’s multiple-comparison test in GraphPad Prism 9 (* *p* < 0.05, ** *p* < 0.01, *** *p* < 0.001, and **** *p* < 0.0001).

To investigate ER stress- and UPR-related signaling pathways, the expression levels of *ire1*, *bip1*, *gpt1*, and *hsp90* were analyzed. Compared with pMS cells, pMS-mapt cells exhibited higher expression of all four genes, with *ire1*, *bip1*, *gpt1*, and *hsp90* expression increased by 11.95-, 2.19-, 5.59-, and 2.52-fold, respectively. Relative to the control pMS cells, vitamin B6-treated pMS-mapt cells exhibited increased expression of all ER stress- and UPR-related genes analyzed, with *ire1*, *bip1*, *gpt1*, and *hsp90* expression elevated by 2.19-, 2.37-, 2.11-, and 3.19-fold, respectively. When compared with untreated pMS-mapt cells, vitamin B6 treatment was associated with lower *ire1* and *gpt1* expression levels, whereas *bip1* and *hsp90* expression levels were further increased. In pMS cells, vitamin B6 treatment resulted in decreased expression of *ire1* (1.10-fold), *bip1* (1.37-fold), and *gpt1* (2.34-fold), whereas *hsp90* expression increased by 1.88-fold compared to untreated pMS cells (Figure 2a,b).

Western blot analysis and subsequent densitometric quantification showed higher IRE1α protein levels in pMS-mapt cells than in pMS cells (Figure 2c). Vitamin B6 treatment produced only minor changes in IRE1α protein expression, resulting in a slight decrease in pMS-mapt cells and a slight increase in pMS cells. In contrast, GRP78 protein levels were reduced in pMS-mapt cells following vitamin B6 treatment.

### 3.3. Effects of Vitamin B6 on Autophagy-Related Gene Expression

**Figure 3:**
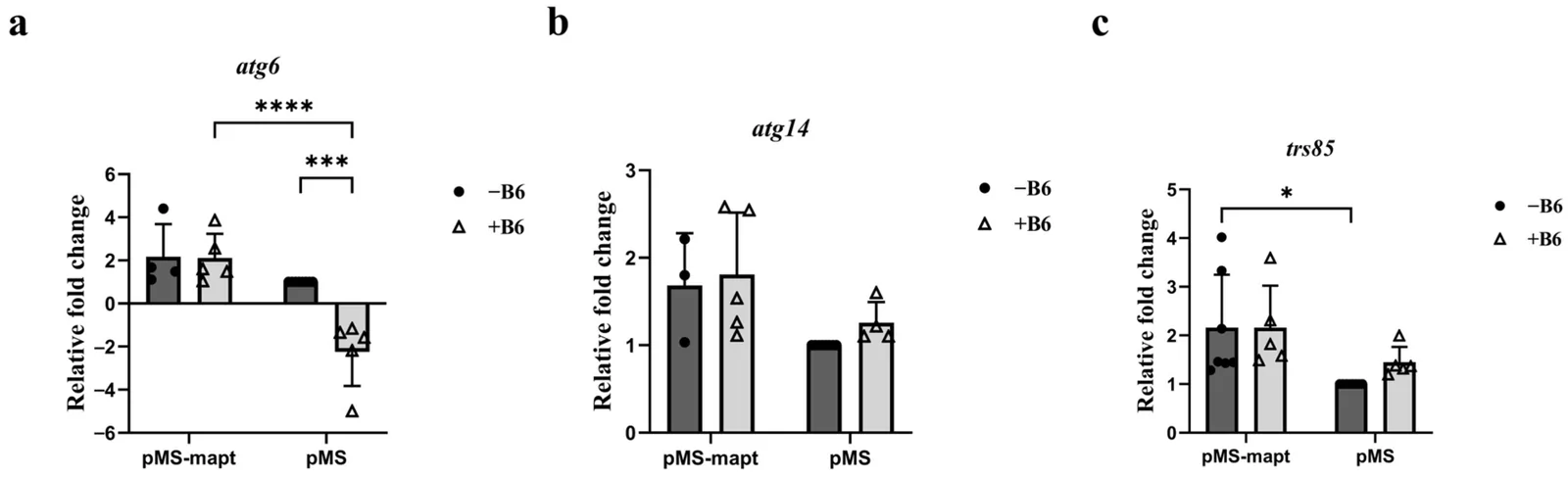
Effects of vitamin B6 on autophagy-related gene expression in pMS and pMS-mapt cells. Relative expression levels of the autophagy-related genes (**a**) *atg6*, (**b**) *atg14*, and (**c**) *trs85* in pMS and pMS-mapt cells in the presence or absence of vitamin B6 treatment. Gene expression levels were normalized to *act1* and are presented relative to untreated pMS cells. Data are shown as mean ± SD from three independent experiments. Statistical analyses were performed using two-way ANOVA followed by Tukey’s multiple comparison test (* *p* = 0.0222, *** *p* = 0.0003, and **** *p* < 0.0001).

To evaluate autophagy-related responses, the expression levels of *atg6*, *atg14*, and *trs85* were analyzed (Figure 3). Relative to the pMS cells, pMS-mapt cells exhibited increased expression of all three genes, with *atg6*, *atg14*, and *trs85* expression elevated by 2.17-, 1.68-, and 2.16-fold, respectively. In vitamin B6-treated pMS-mapt cells, expression levels remained elevated relative to the control group, showing 2.11-, 1.81-, and 2.16-fold increases in *atg6*, *atg14*, and *trs85* expression, respectively. The expression profiles of these genes were similar to those observed in untreated pMS-mapt cells. In pMS cells, vitamin B6 treatment resulted in a 2.25-fold decrease in *atg6* expression, whereas *atg14* and *trs85* expression increased by 1.26- and 1.45-fold, respectively, relative to untreated pMS cells.

### 3.4. Apoptotic Responses in Tau-Expressing Cells

**Figure 4:**
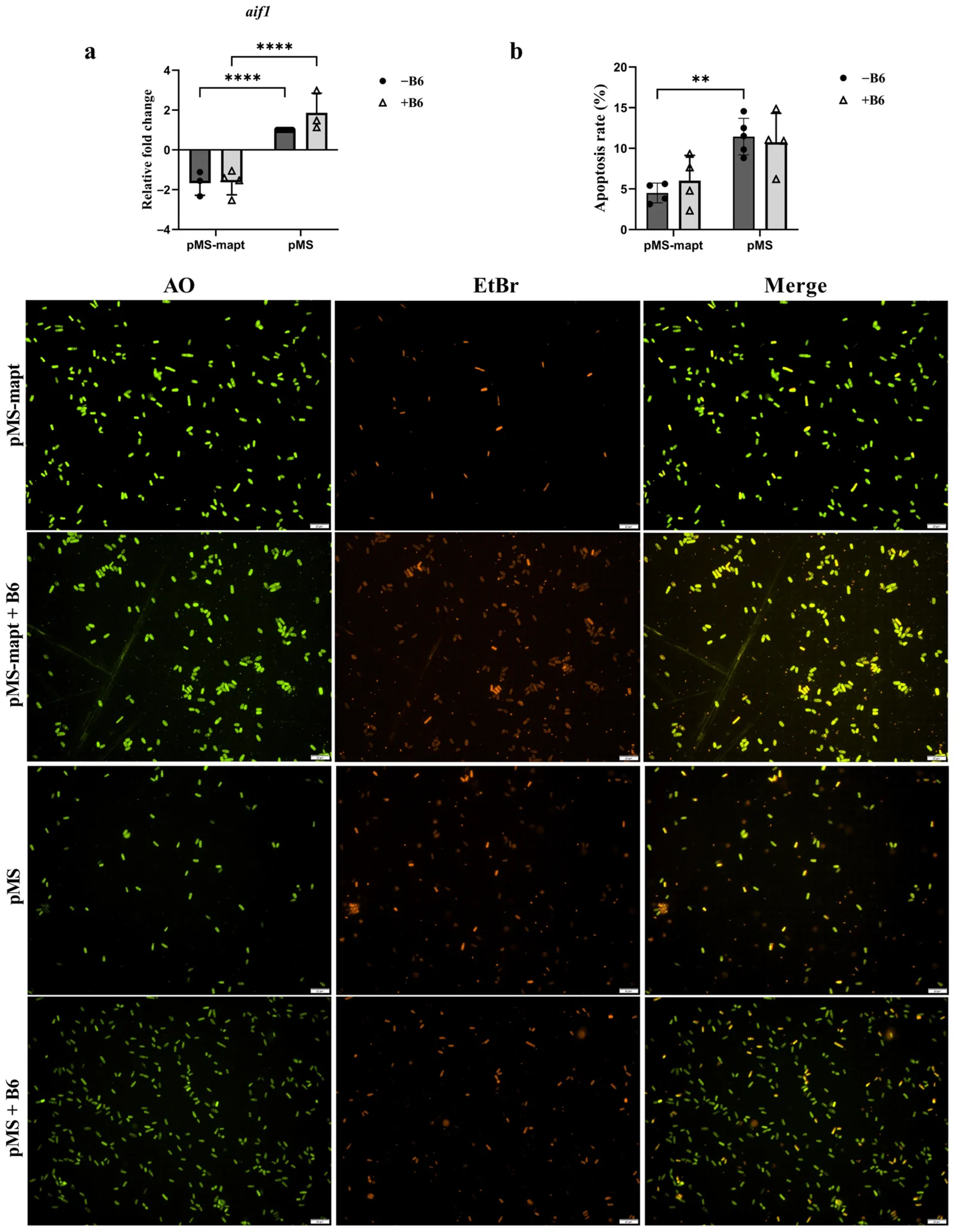
Effects of vitamin B6 on apoptosis-related responses in pMS and pMS-mapt cells. (**a**) Relative expression levels of the apoptosis-related gene *aif1* in pMS and pMS-mapt cells in the presence or absence of vitamin B6 treatment. Gene expression levels were normalized to *act1* and are presented relative to untreated pMS cells. Data are presented as mean ± SD from three independent biological replicates. Statistical significance was determined using two-way ANOVA followed by Tukey’s multiple comparison test (**** *p* < 0.0001). (**b**) Percentage of apoptotic cells determined by AO/EtBr fluorescence staining. Data are presented as mean ± SD from three independent biological replicates. Statistical significance was determined using two-way ANOVA (** *p* = 0.0086). Representative fluorescence microscopy images of untreated and vitamin B6-treated pMS-mapt and pMS cells stained with acridine orange (AO) and ethidium bromide (EtBr). Live cells exhibit green fluorescence, whereas apoptotic and dead cells exhibit orange/red fluorescence. Merged images show the distribution of apoptotic cells under each experimental condition. Scale bar = 20 µm.

Apoptotic responses were assessed by analyzing the *aif1* gene expression and by quantifying apoptotic cells using fluorescence microscopy. Relative to pMS cells, *aif1* expression was lower in pMS-mapt cells. Vitamin B6 treatment did not noticeably alter *aif1* expression in pMS-mapt cells. In contrast, vitamin B6 treatment led to a 1.86-fold increase in *aif1* expression in pMS cells relative to controls (Figure 4a). Apoptotic percentages were determined by fluorescence microscopy using three independent biological replicates. A total of 40 AO-stained and 40 EtBr-stained images were analyzed across all biological replicates. The percentage of apoptotic cells was 4.5% in pMS-mapt cells and 6.03% in vitamin B6-treated pMS-mapt cells. In pMS cells, the apoptotic rate was 11.44% and decreased slightly to 10.76% after vitamin B6 treatment (Figure 4b).

## 4. Discussion

In this study, the effects of vitamin B6 on cellular stress responses associated with tau expression were investigated using a fission yeast model. Tau expression was accompanied by changes in multiple stress-related pathways, including oxidative stress responses, endoplasmic reticulum (ER) stress and unfolded protein response (UPR) signaling, autophagy-related processes, and apoptosis-related responses. Consistent with our previous findings, vitamin B6 treatment reduced *MAPT* expression and tau phosphorylation. In tau-expressing cells, vitamin B6 reduced *ctt1*, *ire1*, and *gpt1* expression, increased *bip1* and *hsp90* expression, and reduced GRP78 protein levels, whereas the examined autophagy- and apoptosis-related responses showed only minor changes.

Tau accumulation and abnormal phosphorylation are closely associated with proteo-toxic stress and disruption of cellular homeostasis. In the present study, vitamin B6 treatment reduced *MAPT* expression, total tau levels, and phosphorylation at the S262, S396, and S404 residues. These phosphorylation sites are located within or near regions that regulate tau interactions with microtubules and have been implicated in the development of tau pathology [13]. Among them, Ser262 phosphorylation has been proposed as an early event in tau dysfunction and disease progression [31]. Therefore, the reduction in tau phosphorylation observed following vitamin B6 treatment may be relevant to the altered stress responses detected in tau-expressing cells.

Tau misfolding and oligomerization are major contributors to cellular toxicity, as tau oligomers impair microtubule stability, intracellular transport, and cellular energy metabolism [32]. Tau aggregation has been shown to reduce neuronal viability and promote cell death in human iPSC-derived neuronal models, with different oligomeric species exhibiting distinct levels of toxicity [33,34]. Similar effects have been reported in yeast systems, where human tau expression is associated with mitochondrial dysfunction and activation of cellular stress pathways [10,35]. Consistent with our previous findings [10], pMS-mapt cells exhibited altered expression of several stress-related genes compared with pMS controls. In the present study, vitamin B6 reduced *MAPT* expression and tau phosphorylation, while also modifying oxidative stress responses, ER stress-associated signaling, autophagy-related gene expression, and apoptosis-related responses. Together, these findings support a close relationship between tau expression and cellular stress pathways and suggest that vitamin B6 was associated with changes in several of the measured stress- and proteostasis-related parameters.

The mechanisms underlying the effects of vitamin B6 on tau phosphorylation remain unclear. The reduction in tau phosphorylation observed in the present study may be related to changes in cellular stress responses and proteostasis pathways, but a direct effect on tau-regulating kinases or phosphatases cannot be excluded. Previous studies have shown that deficiencies in B vitamins can promote tau hyperphosphorylation through changes in kinase and phosphatase activities [36], whereas multivitamin B supplementation reduced tau phosphorylation and improved memory function in a mammalian model [37]. In the present study, vitamin B6 reduced tau phosphorylation; however, cellular function was not directly evaluated. Therefore, whether the observed reduction in tau phosphorylation was accompanied by functional improvement remains unknown. The molecular mechanisms underlying the effect of vitamin B6 on tau phosphorylation also require further investigation.

Untreated pMS-mapt cells exhibited higher *sod1* and *ctt1* expression but lower intracellular ROS production than untreated pMS cells. A similar pattern has been reported in previous studies, in which tau expression was accompanied by increased antioxidant defense responses without a corresponding increase in measurable ROS levels [35,38]. Therefore, the increased expression of *sod1* and *ctt1* may have contributed to the lower ROS signal observed in pMS-mapt cells; however, a direct relationship between the expression of these genes and ROS removal was not examined in the present study. Vitamin B6 increased *sod1* and *ctt1* expression in pMS cells and increased intracellular ROS production in both pMS and pMS-mapt cells. Therefore, the increase in ROS following vitamin B6 treatment was not specific to tau-expressing cells. In pMS-mapt cells, vitamin B6 reduced *ctt1* expression and produced a smaller decrease in *sod1* expression relative to untreated pMS-mapt cells. Despite the increased ROS production, protein carbonyl content did not differ significantly among the experimental groups. These results show that vitamin B6 produced both shared and cell-type-dependent changes in the measured redox-related parameters, without a corresponding significant increase in oxidative protein damage.

Accumulation of misfolded proteins within the ER activates the UPR, a conserved mechanism that helps restore protein homeostasis. Previous studies have shown that pathological tau accumulation can disrupt proteostasis and promote ER stress, leading to activation of UPR signaling pathways [39,40,41]. In *S. pombe*, the ER stress sensor ire1 plays a central role in maintaining ER homeostasis, while bip1, gpt1, and hsp90 contribute to protein folding, quality control, and chaperone-mediated stabilization of proteins [4,42,43,44]. Given the close relationship between tau pathology and proteostasis, changes in the expression of these genes may provide insight into how tau expression and vitamin B6 influence ER stress-related responses.

Compared with pMS cells, untreated pMS-mapt cells exhibited increased expression of *ire1*, *bip1*, *gpt1*, and *hsp90*, together with higher IRE1α protein levels. In pMS-mapt cells, vitamin B6 treatment decreased *ire1* and *gpt1* expression, whereas *bip1* and *hsp90* expression increased relative to untreated pMS-mapt cells. Vitamin B6 also decreased *gpt1* expression and increased *hsp90* expression in pMS cells. Thus, the effects on *gpt1* and *hsp90* were observed in both cell types and were not specific to tau-expressing cells. However, the transcriptional changes were not fully reflected at the protein level. IRE1α protein levels changed only slightly following vitamin B6 treatment, whereas GRP78 protein levels were lower in vitamin B6-treated pMS-mapt cells than in untreated pMS-mapt cells. Overall, vitamin B6 produced both shared and cell-type-dependent transcriptional responses among the ER stress- and UPR-related components examined.

Given the close relationship between ER stress and autophagy, autophagy-related gene expression was also examined. Autophagy plays an important role in the clearance of phosphorylated and aggregation-prone tau species that are relatively resistant to proteasomal degradation [45,46]. In yeast, *atg6* and *atg14* are required for autophagosome formation, whereas *trs85* contributes to selective autophagy through its role in the TRAPPIII complex [47,48,49,50]. In the present study, tau expression was associated with increased expression of *atg6*, *atg14*, and *trs85*, indicating increased expression of the examined autophagy-related genes in pMS-mapt cells. In contrast, vitamin B6 treatment produced only minor changes in the expression of these genes in tau-expressing cells. These findings suggest that the elevated expression of autophagy-related genes is primarily associated with tau expression rather than with vitamin B6 treatment.

Autophagy and apoptosis are closely interconnected cellular processes that contribute to the maintenance of cellular homeostasis under stress conditions. While autophagy generally promotes cell survival through the removal of damaged proteins and organelles, prolonged or dysregulated stress responses may also influence apoptotic signaling pathways [51,52]. This functional relationship has been reported in a variety of model systems, where autophagy- and apoptosis-related pathways act in a coordinated manner to determine cellular fate under proteotoxic stress conditions [53].

In the present study, apoptosis-related responses were evaluated through analysis of *aif1* expression and fluorescence-based quantification of apoptotic cells. AIF1 is a mitochondrial protein associated with caspase-independent cell death and is commonly used as an indicator of mitochondrial stress-related signalling [54]. Both *aif1* expression and apoptotic cell percentages were lower in pMS-mapt cells than in pMS cells, suggesting that a pronounced apoptotic response did not accompany tau expression. Vitamin B6 treatment produced only minor changes in *aif1* expression and apoptotic cell percentages in tau-expressing cells. Together, these findings indicate that the effects of vitamin B6 were more evident in stress-response and proteostasis-related pathways than in apoptosis-related responses.

Overall, the present study shows that tau expression is associated with alterations in multiple cellular stress-response pathways in *S. pombe*. Vitamin B6 treatment was accompanied by changes in oxidative stress responses, ER stress-associated signaling, and other proteostasis-related pathways, together with reduced *MAPT* expression and tau phosphorylation. Although yeast models cannot fully reproduce the complexity of neuronal systems, they provide a useful model system for investigating conserved mechanisms associated with tau-induced cellular stress.

The present study has several limitations. The analyses were restricted to selected transcriptional, protein-level, biochemical, and apoptosis-related parameters measured at a single PLP concentration and treatment time. Changes in the expression of stress- and autophagy-related genes do not directly demonstrate the functional activation of the corresponding pathways. In addition, cytoskeletal organization, autophagic flux, and the subcellular localization of stress- and proteostasis-related proteins were not examined. Therefore, fluorescence-based analysis of these cellular processes will be important for determining how the observed molecular changes are reflected at the cellular level. Further studies in mammalian and in vivo models will be necessary to clarify the molecular basis of vitamin B6 action and to determine the relevance of these findings to neurodegenerative disease.

## Data Availability

All data are available from the author on request.

## Supplementary Materials

The following supporting information can be downloaded at https://www.mdpi.com/article/10.3390/jof12090662/s1, Figure S1: Vitamin B6 reduces tau expression and phosphorylation in *S. pombe*. (a) Relative *MAPT* gene expression levels in *S. pombe* cells expressing human tau (pMS-mapt) in the absence or presence of vitamin B6. Quantitative analysis revealed a significant 2.76-fold reduction in *MAPT* mRNA levels following vitamin B6 treatment. Data are presented as mean ± SD. Statistical significance was determined using Student’s *t*-test (*** *p* < 0.0002). (b) Western blot analysis of phosphorylated tau at Ser262, Ser396, and Ser404 residues, as well as total tau protein levels, in pMS-mapt, pMS-mapt + B6, pMS, and pMS + B6 samples. Vitamin B6 treatment resulted in a marked reduction in tau phosphorylation at all examined sites, concomitant with decreased total tau levels. The asterisk (*) indicates a non-specific band. α-Tubulin was used in normalization.

## Abbreviations

The following abbreviations are used in this manuscript:

ER	Endoplasmic reticulum
UPR	Unfolded protein response
PLP	Pyridoxal 5′-phosphate (PLP)
DCFH-DA	2′,7′-Dichlorofluorescein diacetate
AO	Acridine orange
EtBr	Ethidium bromide
