# Supplementary material for: Vitamin B6 Modifies Tau-Induced Cellular Stress- and Proteostasis-Related Responses
Source: J Fungi (Basel). 2026 Sep 2;12(9):662. doi: 10.3390/jof12090662 (PMC13608788; doi:10.3390/jof12090662)
Supplement: Supplementary file 1 [file jof-12-00662-s001.zip › jof-4489933-supplementary.pdf]

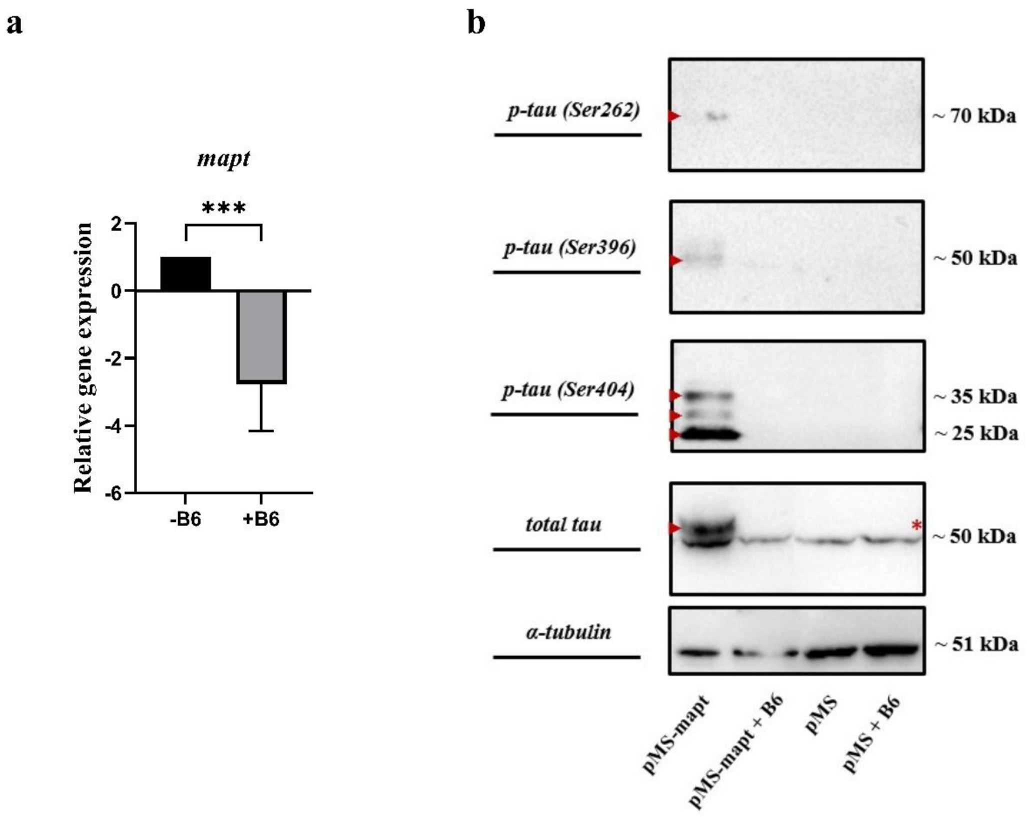

Figure S1. Vitamin B6 reduces tau expression and phosphorylation in *S. pombe*. (a) Relative *MAPT* gene expression levels in *S. pombe* cells expressing human tau (pMS-mapt) in the absence or presence of vitamin B6. Quantitative analysis revealed a significant 2.76-fold reduction in *MAPT* mRNA levels following vitamin B6 treatment. Data are presented as mean ± SD. Statistical significance was determined using Student's *t*-test (***P<0.0002). (b) Western blot analysis of phosphorylated tau at Ser262, Ser396, and Ser404 residues, as well as total tau protein levels, in pMS-mapt, pMS-mapt + B6, pMS, and pMS + B6 samples. Vitamin B6 treatment resulted in a marked reduction in tau phosphorylation at all examined sites, concomitant with decreased total tau levels. The asterisk (*) indicates a non-specific band. α-Tubulin was used in normalization.
